# Eco-friendly synthesis and multifunctional characterization of ZrO_2_/Cu_4_O_3_/MnO_2_/CuMnO_2_ nanocomposite with enhanced thermal stability and antibacterial activity

**DOI:** 10.1186/s11671-026-05003-9

**Published:** 2026-10-14

**Authors:** Adnan Alnehia, M. A. Ahlam, Muhammad Hadi

**Affiliations:** 1 Department of Physics, Faculty of Applied Sciences, Thamar University, Dhamar, 87246 Yemen; 2 Department of Physics, College of Science, Qassim University, Buraydah, 51452 Saudi Arabia; 3 Department of Physics, Faculty of Sciences, King Khalid University, P.O.Box 9004, Abha, Saudi Arabia

**Keywords:** ZrO_2_/Cu_4_O_3_/MnO_2_/CuMnO_2_, Nanocomposite, Bandgap, Antibacterial

## Abstract

**Background:**

This study presents a simple chemical co-precipitation method for synthesizing a multifunctional ZrO2/Cu4O3/MnO2/CuMnO2 nanocomposite, utilizing distilled water as the reaction solvent and an eco-friendly medium, followed by thermal treatment at 500 °C for 2 h. This aqueous-based process allows precise control over the synthesis of a hybrid metal-oxide system, enhancing its thermal and functional properties.

**Methods:**

Comprehensive characterization was performed using X-ray diffraction (XRD), scanning electron microscopy (SEM) coupled with energy dispersive X-ray spectroscopy (EDX), transmission electron microscopy (TEM), thermogravimetric analysis (TGA), Fourier transform infrared spectroscopy (FTIR), and ultraviolet-visible spectroscopy (UV-Vis).

**Results:**

XRD results showed well-crystallized phases with average particle sizes in the nanometer range, while EDX revealed uniform elemental distribution without detectable impurities. The nanocomposite exhibits a direct optical band gap of approximately 2.97 eV and strong absorption in the UV-Vis region, indicating semiconductor behavior. Thermogravimetric analysis confirmed high thermal stability up to elevated temperatures. Additionally, the nanocomposite demonstrated strong antibacterial activity against *Escherichia coli (E. coli)* and *Staphylococcus aureus (S. aureus)*, with inhibition zones reaching up to 18 mm.

**Conclusion:**

The combination of high thermal stability, suitable optical properties, and robust antibacterial performance suggests the potential of this new nanocomposite for applications such as antimicrobial coatings and other multifunctional electronic materials.

## Introduction

Metal oxides constitute a significant group of compounds celebrated for their extraordinary adaptability in a variety of applications due to their unique chemical and physical properties [1–4]. Among these oxides, zirconium dioxide (ZrO_2_), paramelaconite (Cu_4_O_3_), manganese dioxide (MnO_2_), and copper manganese oxide (CuMnO_2_) showcase outstanding structural, optical, and electronic characteristics. Zirconium dioxide is known for its remarkable thermal stability, mechanical strength, wide band gap of ∼5.0 eV, and compatibility with biological systems, making it essential in ceramics, dental materials, and fuel cell technologies. However, its wide band gap limits its optical and photocatalytic activity under visible light [1, 2]. Cu_4_O_3_, notable for its high electrical conductivity, catalytic performance, and a band gap of ∼2.34 eV, is widely used in sensor technology, energy storage systems, and catalytic applications [5]. MnO_2_ is notable for its crucial role in energy storage and environmental remediation due to its redox properties, multiple oxidation states, and a band gap of ∼3.0 eV that facilitates electron transfer processes [6–8]. CuMnO_2_ is a delafossite-type oxide recognized for its p-type conductivity, magnetic properties, and potential in catalysis and spintronic devices [9]. Although individual metal oxides exhibit useful properties, their practical application is often limited by intrinsic drawbacks such as wide band gaps, rapid charge carrier recombination, poor electrical conductivity, aggregation, and limited stability in aqueous or biological environments [10, 11]. To overcome these limitations, the design of multicomponent nanocomposites has emerged as an effective strategy. Combining oxides with different band gap energies, crystal structures, and charge transport behaviors can promote interfacial charge transfer, extend light absorption, and create synergistic effects that are not achievable in single-phase materials [11–15].

In recent years, creating materials made from many different parts at the nanoscale has become a major focus because combining different materials can enhance mechanical strength, thermal stability, and multifunctionality. Studies show that these nanocomposite materials often perform better than the individual components [12–15]. Several hybrid metal oxide systems have been reported, such as CuO-MgO for photocatalysis [16], ZrO_2_/TiO_2_ for supercapacitors [17], and CdO-MgO [18] for antibacterial activity. However, quaternary systems that integrate oxides with complementary properties remain rarely explored. The co-precipitation method stands out as a highly effective approach for producing nanocomposites, providing benefits such as simplicity, cost-effectiveness, and the capability to attain a uniform dispersion of nanoparticles with exact stoichiometry [19, 20]. Other common synthesis routes include sol-gel [21], hydrothermal [22], and solid-state methods [23]. While sol-gel offers good homogeneity and hydrothermal provides good crystallinity, they often require longer reaction times, high pressure, or expensive precursors. In contrast, co-precipitation allows simultaneous precipitation of precursor materials from solution at relatively low temperature, promoting the development of nanostructures essential for enhancing functional properties [19, 20]. The antimicrobial properties of metal oxide nanocomposites have become a key focus, especially given the increasing threat from antibiotic-resistant bacteria [20, 24]. Metal oxide nanocomposites show great potential due to their inherent antibacterial properties, providing a practical approach to address resistant bacterial strains [24, 25]. The scientific rationale for selecting the quaternary ZrO_2_/Cu_4_O_3_/MnO_2_/CuMnO_2_ system lies in the complementary roles of each component: ZrO_2_ provides structural stability and biocompatibility [1, 2], Cu_4_O_3_ and MnO_2_ contribute narrow band gaps for enhanced visible light absorption and redox activity [6–8, 26], and CuMnO_2_ introduces p-type conductivity and magnetic characteristics [9]. The combination of wide-bandgap, narrow-bandgap, and mixed-valence oxides in one matrix is expected to improve charge separation, broaden spectral response, and enhance antibacterial performance compared to reported binary or ternary systems [11, 26]. Despite progress in understanding metal oxide nanocomposites, there is still a lack of thorough studies on the structural, optical, spectral, morphological, and antibacterial characteristics of the ZrO_2_/Cu_4_O_3_/MnO_2_/CuMnO_2_ nanocomposite, especially when synthesized using the co-precipitation method. Addressing this knowledge gap is crucial to fully utilize the potential of these materials. Therefore, this study aims to investigate the structural, optical, spectral, morphological, and antibacterial properties of the ZrO_2_/Cu_4_O_3_/MnO_2_/CuMnO_2_ nanocomposite prepared through the co-precipitation approach. By examining these properties, this research seeks to offer significant insights for developing effective and cost-efficient antibacterial agents that can tackle the challenges of antibiotic-resistant bacteria in real-world scenarios.

## Experimental procedures

### Materials

The synthesis process utilized ZrCl_4_, 97% purity, BDH, MnCl_2_·4H2O, 99% purity, Fluka-Garantie, and CuCl_2_, 98% purity, Sigma-Aldrich as precursor materials. NaOH functioned as a precipitating agent, while distilled water(DW) and ethanol acted as solvents.

### The Synthesis of ZrO_2_/Cu_4_O_3_/MnO_2_/CuMnO_2_ nanocomposite

**Fig. 1:**
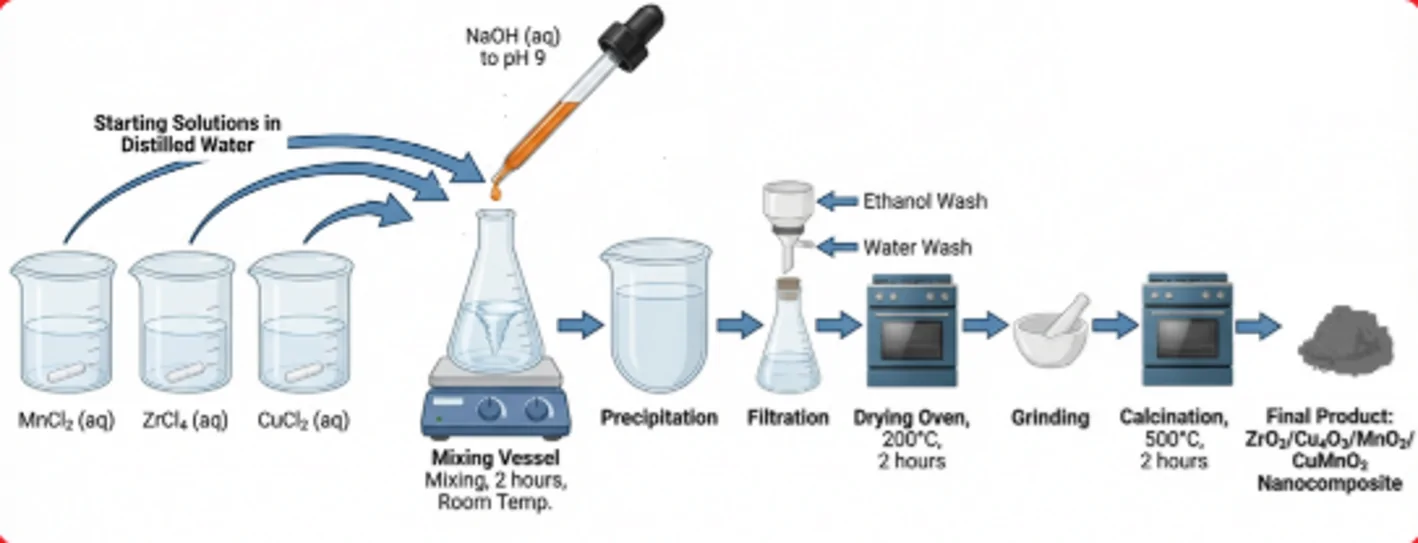
Nanocomposite preparation process flow chart

The preparation process of the ZrO_2_/Cu_4_O_3_/MnO_2_/CuMnO_2_ nanocomposite(NC) was carried out through the following steps: Initially, 2.533 g of MnCl_2_.4H_2_O were dissolved in 20mL of Dw and stirred magnetically for 20 min at 22$\:\pm\:2℃$ until complete homogeneity was achieved. Simultaneously, 3.00 g of ZrCl_4_ and 1.723 g of CuCl_2_ were also dissolved separately under similar conditions to ensure uniform mixing. Subsequently, the three individual solutions were combined into a single beaker and stirred magnetically for two hours at room temperature. In parallel, a solution of 2.4 g of sodium hydroxide (NaOH) in 20 mL of distilled water was prepared and gradually added dropwise to the mixture while stirring, until reaching a pH of 9.After pH adjustment, the resultant solution underwent filtration, and the precipitate obtained was meticulously washed with ethanol and distilled water multiple times to eliminate any contaminants. The precipitate was then dried in an electric oven at 200* ℃* for two hours, followed by grinding into a fine powder and calcination at 500 * ℃* for two hours (see Fig. 1) [25]. Finally, the synthesized NC was stored in a clean container for further analysis and characterization.

### Characterization techniques

The optical properties were assessed using a Hitachi U3900 UV-Vis spectrophotometer (Tokyo, Japan) with Varian Cary-50 software. Measurements were conducted at ambient temperature, covering a wavelength range from 200 to 900 nm. Thermogravimetric analysis (TGA) was carried out on a Mettler Toledo TGA/DSC1 star system (Columbus, OH, USA). Approximately 10 mg of each sample was heated from 25 °C to 1200 °C at a rate of 25 °C per minute in an air atmosphere. X-ray diffraction (XRD) data were collected using an XD–2 X-ray diffractometer with CuK_α_ radiation (λ = 0.154056 nm) operated at 36 kV and 20 mA. Fourier transform infrared (FTIR) spectra were obtained using a Thermo Scientific Nicolet iS10 spectrometer (Madison, WI, USA), employing the KBr pellet method over the range of 500 to 4000 cm⁻¹ with a resolution of 4 cm⁻¹ and accumulating 32 scans per spectrum. Additionally, scanning electron microscopy (SEM) and energy-dispersive X-ray spectroscopy (EDX) analyses were performed using equipment from Jeol Ltd. (Tokyo, Japan).

## Results and discussion

### XRD analysis

**Fig. 2:**
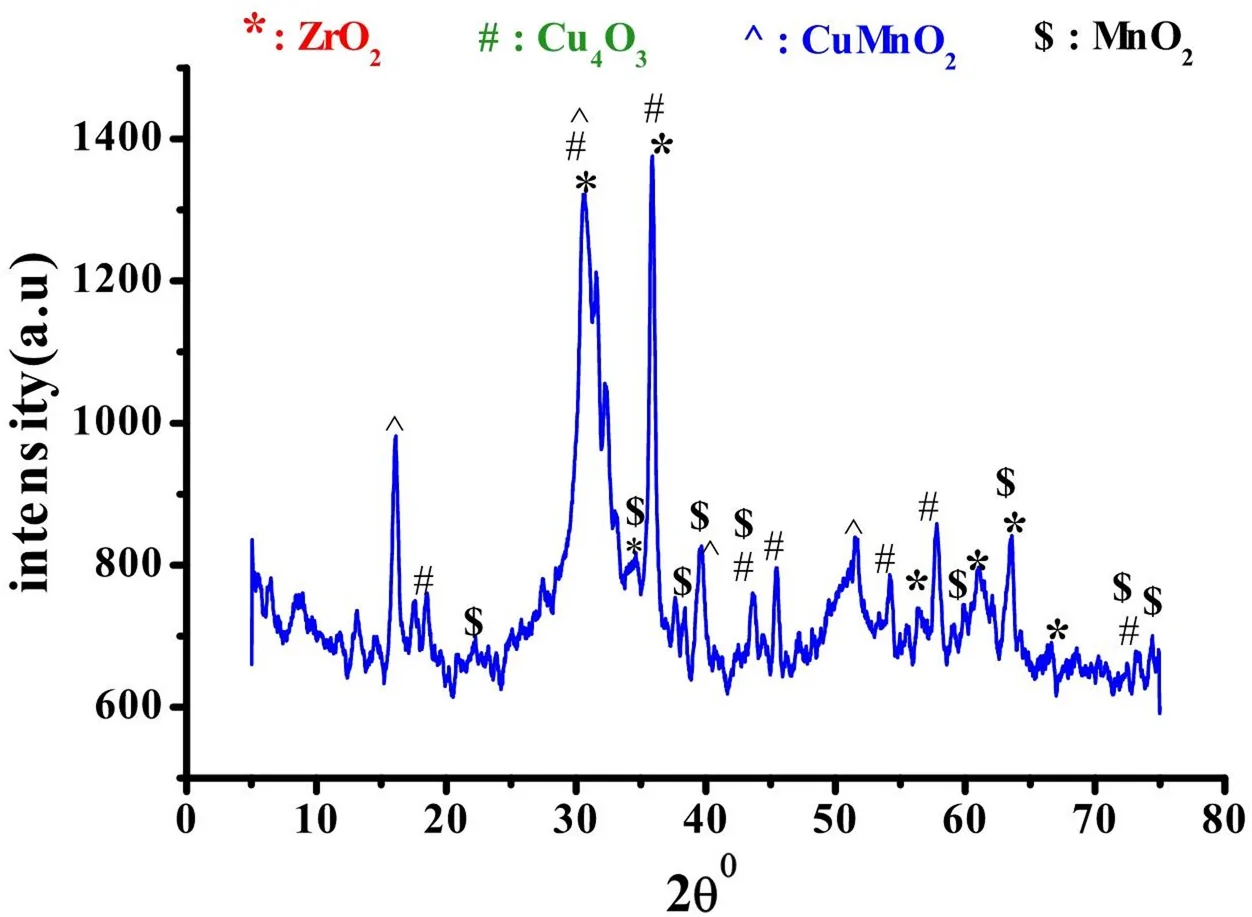
XRD patterns of ZrO_**2**_/Cu_**4**_O_**3**_/MnO_**2**_/CuMnO_**2**_ nanocomposite

The X-ray diffraction (XRD) pattern of the synthesized ZrO₂/Cu₄O₃/MnO₂/CuMnO₂ nanocomposite, following thermal annealing at 500 °C, is presented in Fig. 2.

The observed diffraction maxima were indexed to the orthorhombic phase of ZrO_2_ (space group P212121), the tetragonal structure of Cu_4_O_3_ (space group I41/amd), the orthorhombic MnO_2_ (space group Pnma), and the monoclinic CuMnO_2_ (space group C2/m). The clear resolution of all characteristic peaks for each constituent phase, without detectable impurity or secondary phases, confirms the successful formation of a high-purity, well-crystallized quaternary heterostructure. Phase identification was further substantiated by matching the diffraction angles with standard JCPDS reference cards: No. 34-1084 (ZrO_2_), No. 49-1830 (Cu_4_O_3_), No. 44–0142 (MnO_2_), and No. 50–0860 (CuMnO_2_). The corresponding full-width at half-maximum (FWHM) values and Miller indices for each phase are summarized in Table 1.

**Table 1:** 2 theta, FWHM and Miller indices of the prepared nanocomposite

oxides	2 θ (degree)	θ(degree)	(hkl)	FWHM (degree)
ZrO_2_	30.501	15.250	(111)	0.302
	33.984	16.992	(002)	0.114
	35.899	17.949	(200)	0.290
	53.664	26.832	(202)	0.110
	56.218	28.109	(103)	0.075
	59.003	29.501	(113)	0.130
	60.963	30.481	(311)	0.192
	63.555	31.777	(222)	0.148
MnO_2_	22.172	11.086	(101)	0.069
	34.761	17.381	(301)	0.116
	38.253	19.127	(400)	0.054
	40.095	20.048	(002)	0.174
	43.627	21.814	(401)	0.165
	59.113	29.557	(600)	0.096
	63.077	31.539	(103)	0.093
	72.593	36.297	(221)	0.139
	74.551	37.276	(213)	0.084
Cu_4_O_3_	17.749	8.8745	(101)	0.093
	30.842	15.421	(200)	0.392
	31.581	15.790	(103)	0.148
	35.899	17.949	(202)	0.290
	43.944	21.972	(220)	0.087
	44.350	22.175	(213)	0.065
	58.514	29.257	(224)	0.071
	72.593	36.296	(420)	0.079
CuMnO_2_	16.021	8.0105	(001)	0.237
	31.196	15.598	(002)	0.239
	39.639	19.819	(20 − 2)	0.330
	40.095	20.047	(111)	0.174
	51.650	25.825	(202)	0.056
	59.113	29.556	(31 − 1)	0.096
	73.478	36.739	(022)	0.169
	74.551	37.275	(-221)	0.084

To estimate the average crystallite size (D) of the nanocomposite, the Debye–Scherrer formula was applied (Eq. (1)), where λ = 0.15406 nm, θ is the Bragg angle, and β is the FWHM [27].

1 $$\:D=\frac{0.9\lambda\:}{\beta\:cos\theta\:}$$

Concurrently, the dislocation density (δ), serving as an indicator of lattice imperfections, was calculated using Eq. (2) [28].

2 $$\:{\updelta\:}=\frac{1}{{D}^{2}}$$

Additionally, both crystallite size and microstrain (ε) were derived from the Williamson–Hall (W–H) method, as expressed in Eq. (3) [29]. The calculated average crystallite sizes are documented in Table 2.

3 $$\:\beta\:\mathrm{c}\mathrm{o}\mathrm{s}\theta\:=0.9\lambda\:/D+4\epsilon sin\theta\:\:$$

For the orthorhombic ZrO_2_ phase, the Scherrer estimate yielded a crystallite size of approximately 67.47 nm, whereas the W–H analysis, which accounts for strain-induced broadening, gave a notably lower value of 23.11 nm. The retention of the metastable orthorhombic polymorph at ambient temperature, instead of the thermodynamically favored monoclinic form, is attributable to two primary influences: (i) the nanosize effect, which tends to stabilize higher-symmetry phases below a critical threshold of ~ 30 nm, and (ii) interfacial strain generated by the close contact between ZrO_2_ and the other oxide components within the composite. Such local orthorhombic distortions driven by size and strain effects have been corroborated by recent pair distribution function studies [30].

**Table 2:** Average *D*(nm) and dislocation density of the prepared nanocomposite

Oxides	average D(nm)	average dislocation density(lines/m^2^)*10^14^	average lattice strain ($\:\boldsymbol{\epsilon\:}$)	average D(nm)	average dislocation density(lines/m^2^)*10^14^	lattice strain ($\:\boldsymbol{\epsilon\:}$)
	by Scherer method			by Williamson Hall (W-H) method		
ZrO_2_	67.47	2.197	0.000679	23.11	18.7241	-0.00219
Cu_4_O_3_	89.13	1.259	0.000631	30.28	10.9066	-0.00163
MnO_2_	92.30	0.391	0.000244	72.20	1.91751	-0.000116
CuMnO_2_	75.58	1.751	0.000695	84.08	1.414540	-0.00174

A notable divergence between Scherrer- and W–H-derived crystallite sizes was observed, particularly for ZrO_2_ and Cu_4_O_3_. This discrepancy arises because the Scherrer equation attributes peak broadening solely to crystallite dimensions, neglecting lattice strain. In contrast, the W–H method decouples size and strain contributions. The significantly smaller W–H sizes, together with relatively high negative microstrain values (− 0.00219 for ZrO_2_ and − 0.00163 for Cu_4_O_3_), indicate substantial compressive strain within the composite matrix. Such strain is likely induced by the coexistence of four distinct metal oxides with differing ionic radii and crystal symmetries, promoting lattice distortion and defect formation. Hence, the W–H approach offers a more reliable crystallite size estimation for this multicomponent system. Conversely, MnO_2_ exhibited comparable sizes from both methods, consistent with its minimal strain contribution. The microstrain data compiled in Table 2 reveal appreciable lattice strain throughout the nanocomposite, reflecting internal stresses arising from phase heterogeneity and interfacial interactions. Lattice strain can exert dual effects on material performance: moderate strain may enhance functional properties, such as electrical or ionic conductivity, by introducing additional active sites, which is advantageous for catalytic and energy-storage applications; however, excessive strain can compromise structural coherence and mechanical stability. Therefore, quantifying lattice strain is essential for rational synthesis optimization and property tailoring. The negative strain values confirm the prevalence of compressive strain, commonly observed in nanocomposites due to lattice mismatch and nanoscale surface tension. Such compressive strain not only improves structural robustness but may also modulate the electronic band structure and defect concentration.

### FTIR analysis

**Fig. 3:**
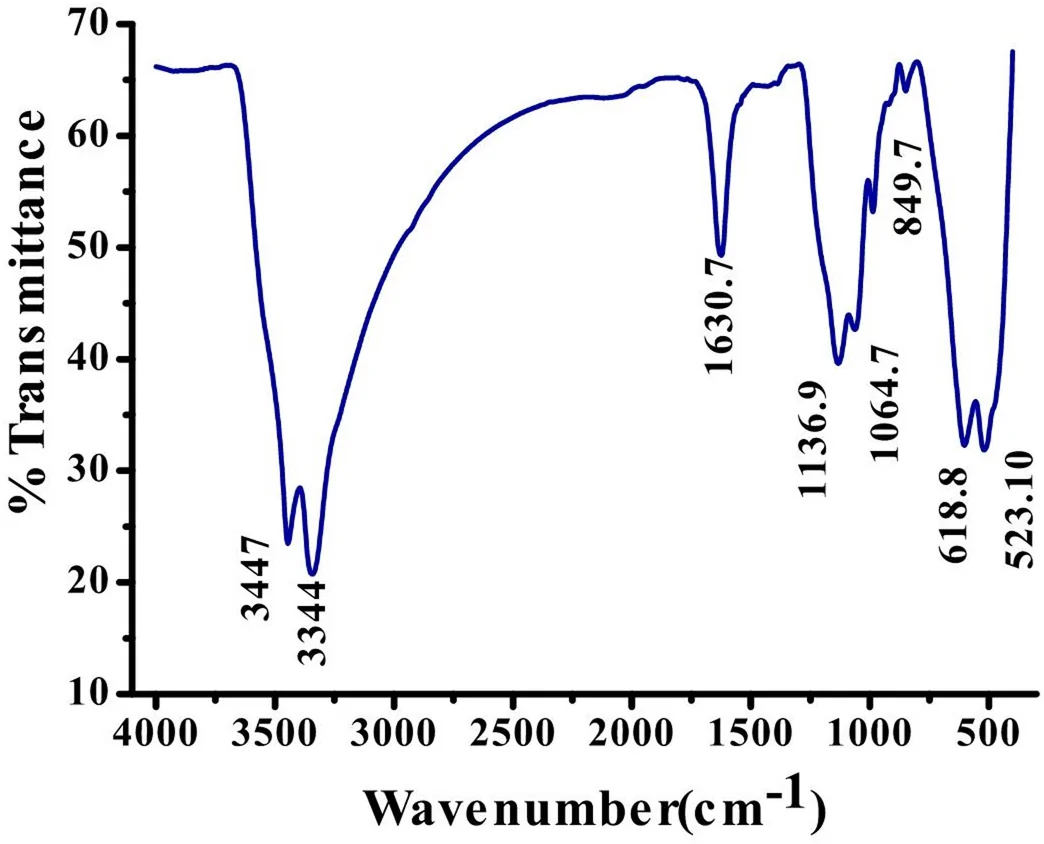
FTIR spectra of ZrO_**2**_/Cu_**4**_O_**3**_/MnO_**2**_/CuMnO_**2**_ nanocomposite

FTIR spectroscopy was used to examine the chemical environment of the surface and to verify the formation of metal–oxygen linkages in the ZrO_2_/Cu_4_O_3_/MnO_2_/CuMnO_2_ nanocomposite after thermal treatment at 500 °C. It should be clarified that FTIR identifies bond types and adsorbed species, but it cannot independently establish crystalline phase identity. Phase identification relies on XRD. The spectrum presented in Fig. 3 exhibits three characteristic regions. At high wavenumbers, the broad envelope at 3447 cm⁻¹ with an inflection at 3344 cm⁻¹ originates from O–H stretching of adsorbed water and surface hydroxyls. The band at 1630.7 cm⁻¹ corresponds to the bending mode of coordinated water. These features are expected for high-surface-area oxides produced via aqueous co-precipitation, where exposure to air leads to surface hydration even after calcination [31, 32]. Between 1200 and 800 cm⁻¹, three bands are resolved at 1136.9 cm⁻¹, 1064.7 cm⁻¹, and 849.7 cm⁻¹. A key point in interpreting transition metal oxides is that the primary M–O lattice stretching modes appear below 1000 cm⁻¹. Therefore, features above this limit should not be attributed to direct lattice metal–oxygen vibrations. In systems prepared from metal chlorides and precipitated with NaOH, bands in the 1100–1050 cm⁻¹ interval are commonly associated with surface-bound residues such as carbonate species and with hydroxyl deformation modes that survive decomposition of precursors [33]. This interpretation aligns with reports on ternary MnO_2_/CuO/ZrO_2_ composites, where absorptions in the 1000–1200 cm⁻¹ range were assigned to surface adsorbed species [33]. The band at 849.7 cm⁻¹ sits at the lower edge of this region and is best described as arising from M–O–M bridging vibrations formed during condensation of the hydroxide intermediates [34, 35]. The region below 1000 cm⁻¹ contains the most structurally informative bands. Two clear absorptions at 618.8 cm⁻¹ and 523.1 cm⁻¹ are observed. These wavenumbers are consistent with M–O stretching and O–M–O bending vibrations within oxide lattices, and they support the conclusion that metal–oxygen frameworks formed during calcination. Because the present material contains Zr, Cu, and Mn in multiple oxidation states, the vibrational modes of ZrO_2_, Cu_4_O_3_, MnO_2_, and CuMnO_2_ will overlap heavily in this region. As a result, FTIR cannot be used to deconvolute these contributions and assign individual peaks to specific metal–oxygen bonds or to specific phases. That level of assignment requires diffraction data [7, 25, 34, 36, 37].

### SEM and EDX analysis

**Fig. 4:**
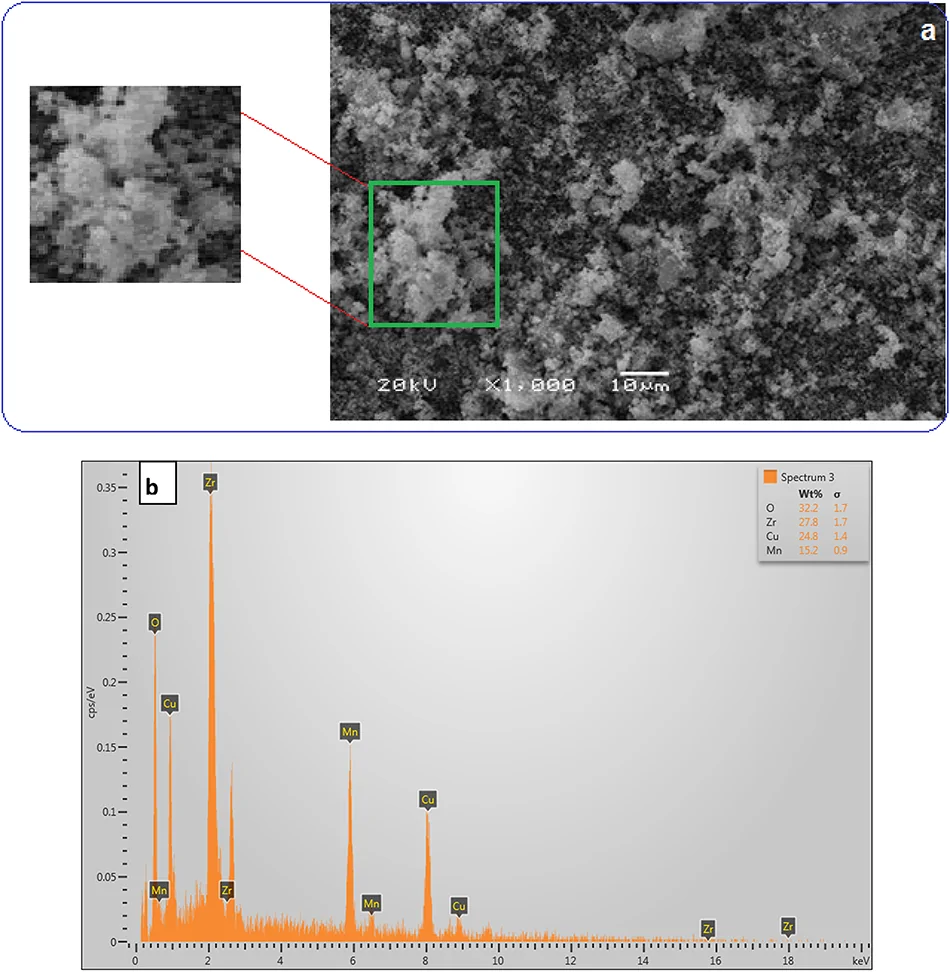
(**a**) SEM image (**b**) EDX analysis of ZrO_2_/Cu_4_O_3_/MnO_2_/CuMnO_2_ nanocomposite

The surface morphology of the synthesized ZrO_2_/Cu_4_O_3_/MnO_2_/CuMnO_2_ nanocomposite, prepared via the co-precipitation method at 500 °C for 2 h, was examined using SEM. The SEM micrograph, presented in Fig. 4a, reveals a heterogeneous surface topography characterized by a wide distribution of particle sizes and irregular shapes. The microstructure consists of agglomerated clusters of fine primary particles, interspersed with larger, roughly spherical secondary particles. This irregular and non-uniform morphology is indicative of the complex nucleation and growth processes occurring during the co-precipitation and subsequent calcination steps, which involve the formation of multiple oxide phases simultaneously. The lack of well-defined, faceted crystallites suggests a relatively rapid growth rate, potentially hindered by the presence of different constituent elements, which can lead to the formation of less-ordered structures. To confirm the elemental composition and chemical purity of the synthesized nanocomposite, energy-dispersive X-ray (EDX) spectroscopy was performed. The corresponding EDX spectrum, shown in Fig. 4b, clearly displays the characteristic peaks for the constituent elements: manganese (Mn), zirconium (Zr), oxygen (O), and copper (Cu). No other elemental peaks were detected within the detection limit of the EDX system, confirming the high purity of the prepared nanocomposite and the absence of any significant contaminants from the synthesis process. The semi-quantitative elemental composition, as calculated from the EDX analysis, is indicated in the figure, providing further validation of the intended stoichiometry of the nanocomposite.

### TEM analysis

**Fig. 5:**
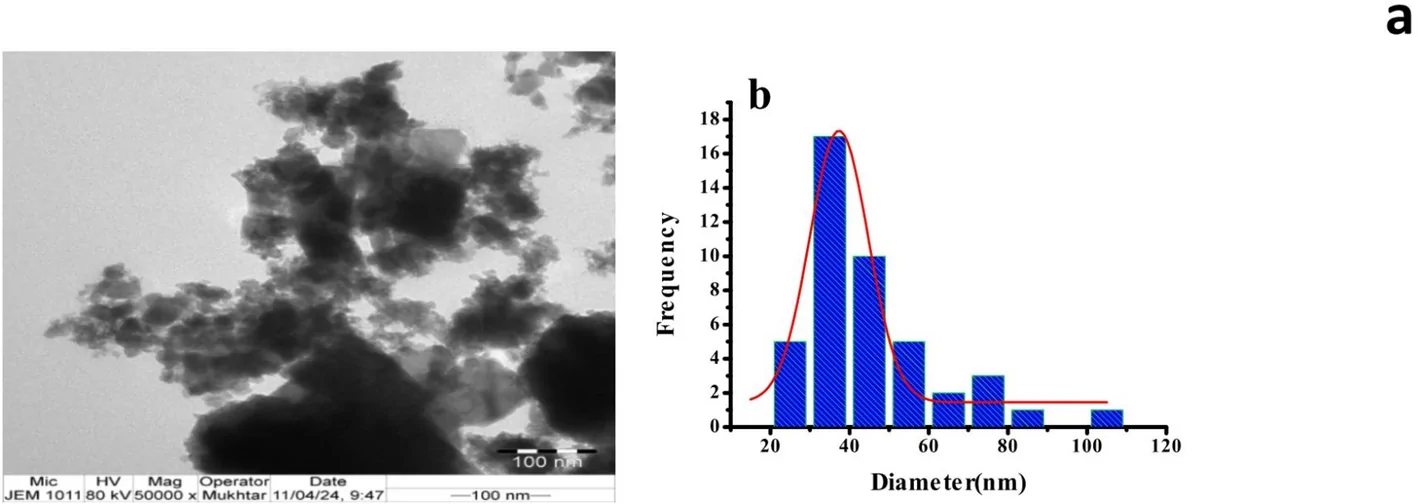
(**a**) TEM image and (**b**) the corresponding histogram of the particle size distribution

TEM was employed to provide a more detailed insight into the morphology, crystallinity, and particle size distribution of the synthesized nanocomposite. Figure 5a displays a representative TEM micrograph of the ZrO_2_/Cu_4_O_3_/MnO_2_/CuMnO_2_ nanocomposite. The image reveals a complex and diverse morphology, consistent with the SEM observations. The nanostructure consists of a mixture of distinctly different particle shapes, including a significant population of nearly spherical nanoparticles, alongside a notable fraction of polyhedral and highly irregular-shaped particles. The edges of the particles are generally not sharp, suggesting a degree of surface roughness at the nanoscale. Furthermore, the TEM image shows a high degree of particle agglomeration, which is a common characteristic of nanostructured materials synthesized via wet-chemical routes due to their high surface energy and the tendency to minimize it. The corresponding particle size distribution histogram, derived from measuring over 100 particles in multiple TEM images (Fig. 5b), shows a relatively broad size distribution, which is expected given the multiphasic composition of the nanocomposite. The calculated average particle size is 38.50 nm. The presence of orthorhombic ZrO_2_, tetragonal Cu_4_O_3_, and the mixed metal oxides MnO_2_ and CuMnO_2_, each with different crystal structures (tetragonal, hexagonal, etc.) and surface energies, dictates the final size and shape of the resulting nanoparticles. The formation of such a polydisperse system further confirms the complexity of the synthesis pathway and the successful integration of multiple components into a single nanocomposite.

It is important to distinguish between crystallite size obtained from XRD and particle size observed by TEM. The average particle size from TEM analysis was found to be 38.50 nm, while the crystallite size from XRD using the Scherrer method reached up to 92.30 nm for MnO_2_. This difference is expected because XRD measures the size of a single coherently diffracting crystalline domain, whereas TEM measures the physical size of the particle as it appears in the image. In many cases, a single particle observed in TEM can be composed of several aggregated crystallites. The high-temperature calcination at 500 °C used in this study likely promoted the agglomeration of smaller crystallites into larger secondary particles. Therefore, the TEM size represents the agglomerated particle size, while the XRD size represents the size of the individual crystallites within those particles. This behavior is commonly reported in multi-component metal oxide nanocomposites.

### TGA analysis

**Fig. 6:**
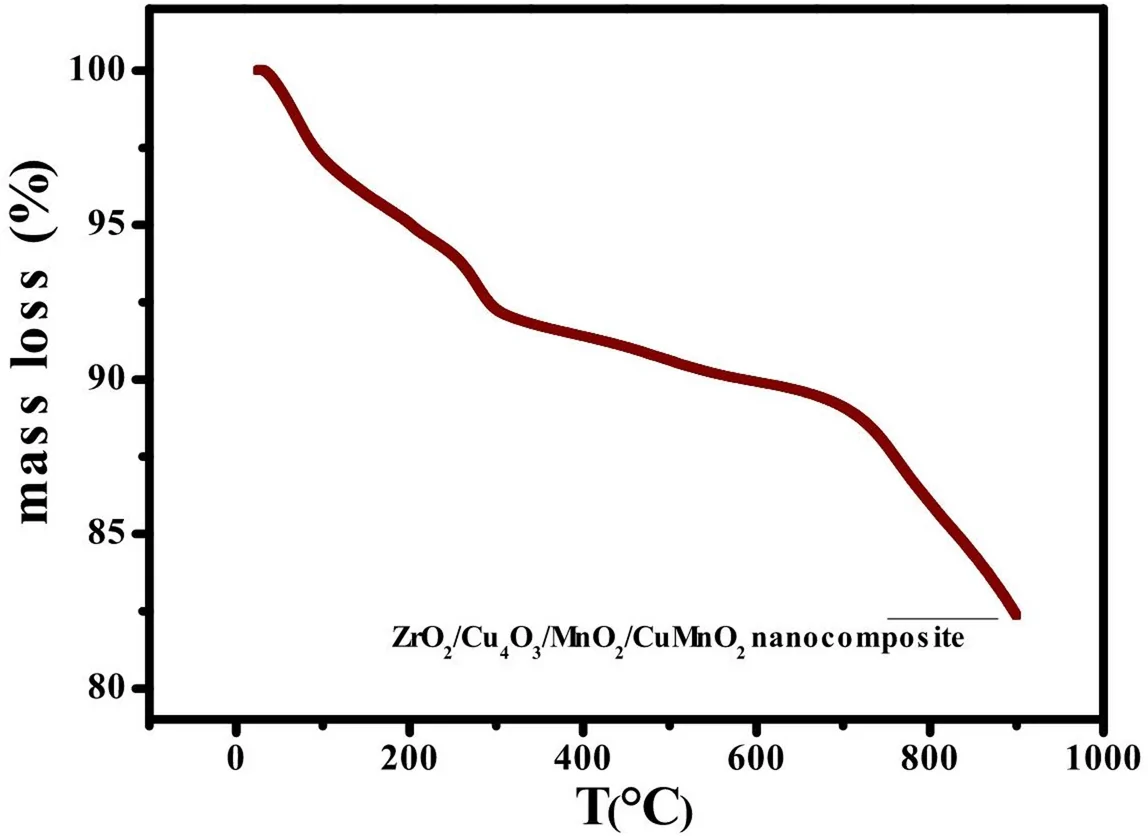
TGA analysis of ZrO_2_/Cu_4_O_3_/MnO_2_/CuMnO_2_ nanocomposite

The TGA curve of the ZrO_2_/Cu_4_O_3_/MnO_2_/CuMnO_2_ nanocomposite prepared by co-precipitation using only distilled water, shown in Fig. 6, exhibits complex thermal behavior reflecting its multiple components and their interactions with the gradual increase in temperature from room temperature up to 1000 °C.The curve begins with a slight weight loss of approximately 4% in the initial stage from room temperature to about 250 °C. This loss is mainly attributed to the evaporation of surface moisture and physically adsorbed water on the surface of the nanoparticles, a phenomenon common in oxide nanomaterials due to their large surface area [38].Subsequently, the compound exhibits a period of thermal stability from approximately 250 °C to about 500 °C, during which the curve is almost flat with negligible weight loss. This indicates the absence of residual organic solvent impurities, consistent with the use of distilled water as the sole solvent. However, upon exceeding 500 °C, the most critical stage of the analysis begins. The curve shows a significant weight loss of 8% continuing until approximately 800 °C, followed by an additional loss of 4% from 800 °C to 900 °C. These losses are mainly attributed to the thermal decomposition of manganese dioxide (MnO_2_) and its conversion to manganese trioxide (Mn_2_O_3_) with the release of oxygen gas, followed by possible further transformations (e.g., Mn_2_O_3_ to Mn_3_O_4_) or the formation of new phases such as copper-manganese spinel (CuMn_2_O_4_). The experimental weight loss matches the theoretical values expected for these reactions, confirming the presence of a large amount of MnO_2_ in the sample. As the temperature approaches 1000 °C, the curve begins to stabilize again, indicating the formation of thermally stable terminal phases [39, 40] .Based on this analysis, it can be concluded that the nanocomposite exhibits only limited thermal stability up to 500 °C. Above this temperature, the material undergoes progressive decomposition and phase transformations and does not become fully stable again until approximately 1000 °C. Therefore, the nanocomposite is suitable for applications operating only below 500 °C, but its use is limited in applications requiring prolonged exposure to temperatures exceeding 500 °C.

### UV-visible analysis

**Fig. 7:**
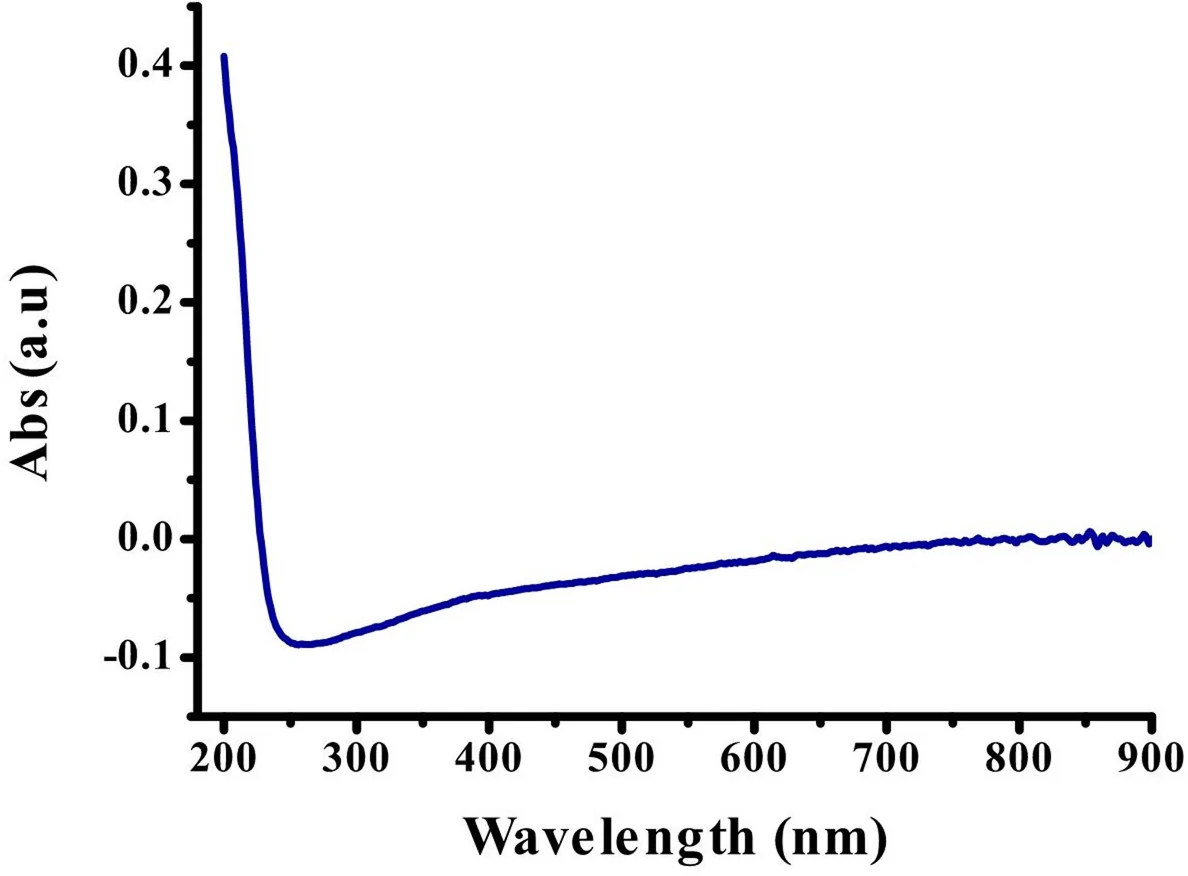
Absorption spectra of ZrO_2_/Cu_4_O_3_/MnO_2_/CuMnO_2_ nanocomposite

The optical characteristics of the ZrO_2_/Cu_4_O_3_/MnO_2_/CuMnO_2_ quaternary nanocomposite were investigated using UV-Visible spectroscopy in transmission mode. The spectra were recorded for a colloidal dispersion in a standard 1 cm quartz cell across 200–900 nm, as illustrated in Fig. 7. A pronounced absorption was observed in the ultraviolet region, which progressively declined into the visible range. The spectrum exhibits a broad absorption profile without well-defined peaks. This feature can be ascribed to the complex multi-component structure of the nanocomposite, where the electronic transitions of ZrO_2_, Cu_4_O_3_, MnO_2_, and CuMnO_2_ overlap, leading to a continuous absorption band [10, 41].

**Fig. 8:**
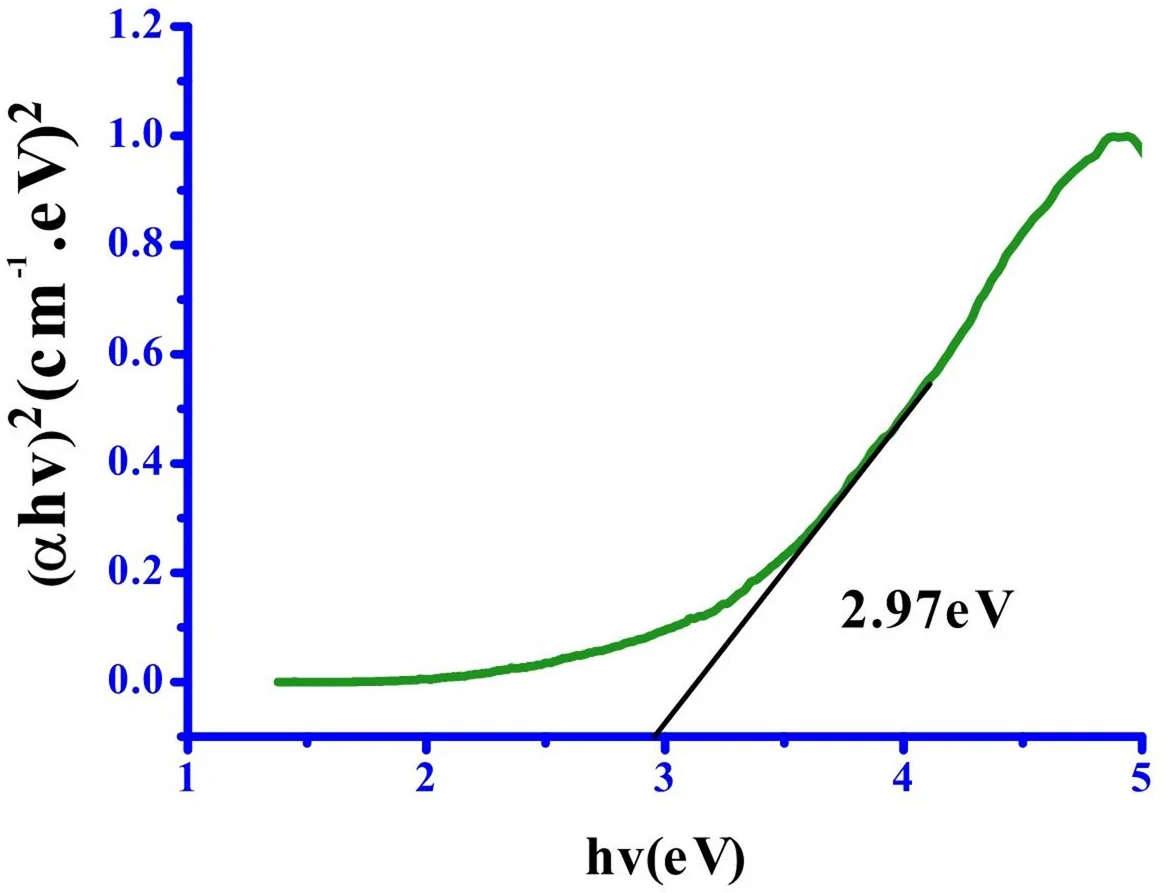
Optical band gap of ZrO_2_/Cu_4_O_3_/MnO_2_/CuMnO_2_ nanocomposite

The direct optical band gap was extracted using Tauc’s approach for direct allowed transitions, expressed as (αhv)^2^ = A(hv - Eg) [42, 43]. The absorption coefficient(α), was derived from the absorbance values based on the Beer-Lambert law. The Tauc plot, presented in Fig. 8, gives a direct band gap of 2.97 eV for the nanocomposite. This value is lower than those reported for pure ZrO_2_ (5.0 eV), and lies between the values of Cu_4_O_3_ (2.34 eV), MnO_2_ (1.2–2.3 eV), and CuMnO_2_ (1.6 eV). The narrowing of the band gap relative to ZrO_2_ confirms the successful formation of a heterostructure, which enhances photon harvesting in the visible region [1, 2, 5–9].

Such a band gap in the visible range suggests that the prepared ZrO_2_/Cu_4_O_3_/MnO_2_/CuMnO_2_ nanocomposite can be activated by solar light. Therefore, it shows potential for application in solar-driven photocatalysis for environmental remediation.

### Antibacterial activity

**Fig. 9:**
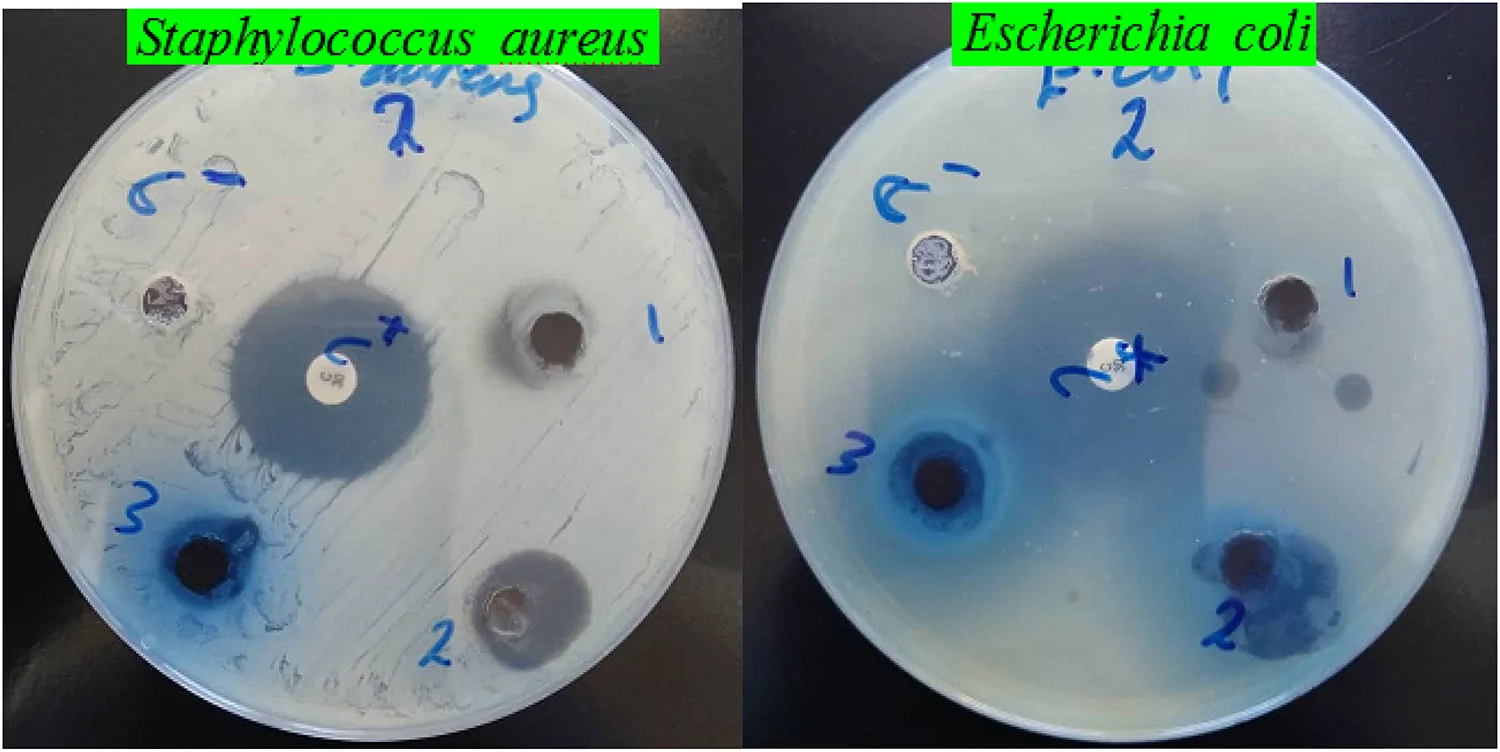
Antibacterial activity of ZrO2/Cu4O3/MnO2/CuMnO2 nanocomposite against S. aureus and E.coli. (1) 50 mg/ml (2) 100 mg/ml (3) 200 mg/ml, (C−) d.H2O as the negative control and(C+) Azithromycin (AZM) as the positive control

**Fig. 10:**
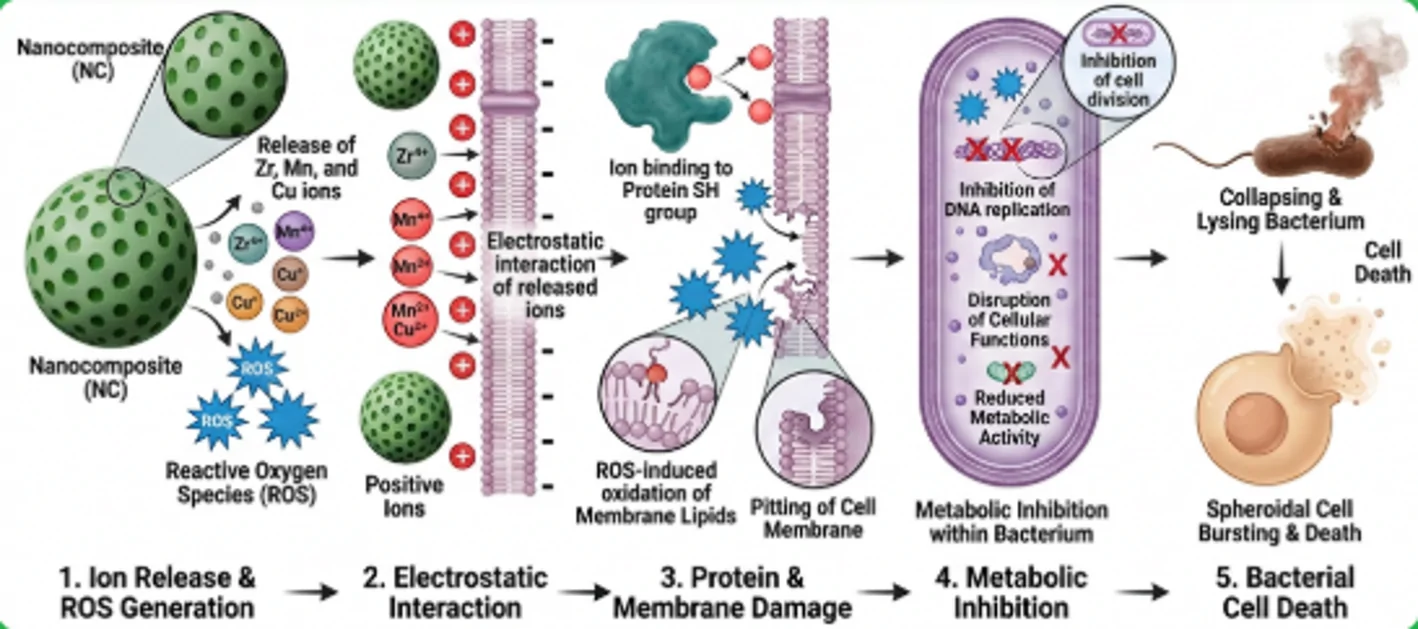
Antibacterial Activity Mechanism of the ZrO_2_/Cu_4_O_3_/MnO_2_/CuMnO_2_Nanocomposite

The growth-inhibitory potential of the quaternary nanocomposite ZrO_2_/Cu_4_O_3_/MnO_2_/CuMnO_2_ was assessed using the agar well diffusion method against two representative bacterial strains: Gram-positive *Staphylococcus aureus*(*S. aureus*) and Gram-negative *Escherichia coli* (*E.coli*).Bacterial suspensions were prepared and uniformly spread onto Mueller-Hinton agar plates. Filter paper discs, 6 mm in diameter, were impregnated with nanocomposite dispersions at concentrations of 50, 100, and 200 mg/mL prepared in deionized water. The loaded discs were placed on the inoculated plates and incubated at 37 °C for 22 h. Following incubation, the diameters of the zones of inhibition ZOI were measured. All experiments were performed in duplicate *n* = 2 [25, 44].The average ZOI values are presented in Table 3. The nanocomposite exhibited inhibitory effects against both strains, with comparatively larger inhibition zones observed for *E. coli* than for *S. aureus*, as illustrated in Fig. 9 .It should be noted that the disc diffusion assay provides evidence of growth suppression only and is not sufficient to confirm whether the effect is bacteriostatic or bactericidal. Further investigations, including determination of the minimum inhibitory concentration MIC, minimum bactericidal concentration MBC, and time-kill kinetics, would be required to clarify the nature of the antibacterial action.The underlying mechanism responsible for the observed inhibition remains undefined in the current work and is therefore proposed as hypothetical. The effect may plausibly arise from a combination of factors: (a) release of metal ions from the nanocomposite surface, (b) direct physical interaction between high-surface-area nanoparticles and bacterial cells, and (c) possible generation of reactive oxygen species ROS [32, 38, 45, 46].Metal ions that could potentially be released include Mn⁴⁺, Mn²⁺, Zr⁴⁺, Cu⁺, and Cu²⁺. Their propensity for leaching in aqueous media is expected to vary. Cu²⁺ and Mn²⁺ are more likely to detach from the crystal lattice, whereas Zr⁴⁺ and Mn⁴⁺ are anticipated to remain largely embedded within the oxide framework and to be released only to a limited extent under specific conditions, such as acidic environments [29, 31, 38] .In general, positively charged ions may interact electrostatically with anionic components of the bacterial envelope and with thiol -SH groups in essential proteins, potentially leading to membrane destabilization [42]. A schematic representation of this proposed interaction is provided in Fig. 10. Given the hypothetical nature of the proposed mechanism, follow-up studies are warranted. These should include direct quantification of ROS, measurement of metal ion release under physiologically relevant conditions, and assessment of membrane integrity using fluorescent probes or electron microscopy.Regarding the apparent difference in susceptibility between the two strains, no conclusive explanation can be drawn from the present data. It is recognized that Gram-negative bacteria possess an additional outer membrane that may restrict penetration, while Gram-positive bacteria have a thicker peptidoglycan layer. Considering the interplay of these structural features along with other variables such as growth rate and surface composition, any interpretation of the observed difference remains preliminary and requires targeted comparative studies [47, 48]. The significance of these preliminary findings lies in providing initial evidence that the synthesized quaternary nanocomposite possesses measurable growth-inhibitory activity against representative bacterial species. The multi-component design may offer the possibility of synergistic effects not observed with single-phase oxides. Accordingly, this study establishes a foundation for subsequent research to explore effective dosages, detailed mechanisms, and potential applications in areas such as antibacterial surface coatings and environmental decontamination.

**Table 3:** Antibacterial properties of ZrO_2_/Cu_4_O_3_/MnO_2_/CuMnO_2_ at different concentrations

Name of bacteria	Zone of inhibition (ZOI; diameter in mm) ± standard deviation			
	50 mg/ml	100 mg/ml	200 mg/ml	Azithromycin (control)
*S. aureus*	$\:8\pm\:0.75$	$\:10\pm\:0.25$	$\:12\pm\:0.50$	$\:25\pm\:0.50$
*E.coli*	-	$\:8\pm\:0.75$	$\:18\pm\:0.25$	$\:30\pm\:0.75$

## Conclusion

In conclusion, the ZrO_2_/Cu_4_O_3_/MnO_2_/CuMnO_2_ nanocomposite was successfully synthesized using an aqueous co-precipitation method. The use of distilled water as the reaction solvent makes the process environmentally friendly. Characterization revealed well-crystallized phases with an average crystallite size in the nanometer range, confirming a high surface area. The material exhibited a direct optical band gap of 2.97 eV and excellent thermal stability up to elevated temperatures, as shown by TGA analysis. Furthermore, the nanocomposite demonstrated significant antibacterial activity, with an inhibition zone of up to 18 mm against *E. coli*, indicating its potential for antimicrobial coating applications. These combined properties of thermal stability, suitable optical characteristics, and antibacterial performance suggest that the synthesized nanocomposite is a promising candidate for multifunctional material applications. Future research will focus on investigating the antibacterial mechanisms in detail and exploring the material’s performance in specific device configurations to further advance its practical applications in industrial and biomedical fields.

## Data Availability

All data generated or analyzed during this study are included in this manuscript.

## Declarations

### Dual publication

This manuscript is not under consideration for publication elsewhere.
